# Sensitization of Cancer Cells through Reduction of Total Akt and Downregulation of Salinomycin-Induced pAkt, pGSk3*β*, pTSC2, and p4EBP1 by Cotreatment with MK-2206

**DOI:** 10.1155/2014/295760

**Published:** 2014-07-08

**Authors:** Ae-Ran Choi, Ju-Hwa Kim, Sungpil Yoon

**Affiliations:** Research Institute, National Cancer Center, Goyang-si, Gyeonggi-do 411-764, Republic of Korea

## Abstract

MK-2206 is an inhibitor of Akt activation. It has been investigated as an anticancer drug in clinical trials assessing the potential of pAkt targeting therapy. The purpose of this study was to identify conditions that increase the sensitivity of cancer cells to MK-2206. We found that the treatment of cancer cells with a high concentration of salinomycin (Sal) reduced total Akt protein levels but increased activated Akt levels. When cancer cells were cotreated with MK-2206 and Sal, both pAkt and total Akt levels were reduced. Using microscopic observation, an assessment of cleaved PARP, FACS analysis of pre-G1 region, and Hoechst staining, we found that Sal increased apoptosis of MK-2206-treated cancer cells. These results suggest that cotreatment with MK-2206 and Sal sensitizes cancer cells via reduction of both pAkt and total Akt. Furthermore, cotreatment of cancer cells with Sal and MK-2206 reduced pp70S6K, pmTOR, and pPDK1 levels. In addition, Sal-induced activation of GSK3*β*, TSC2, and 4EBP1 was abolished by MK-2206 cotreatment. These results suggest that cotreatment using MK-2206 and Sal could be used as a therapeutic method to sensitize cancer cells through targeting of the PI3K/Akt/mTOR pathway. Our findings may contribute to the development of MK-2206-based sensitization therapies for cancer patients.

## 1. Introduction

MK-2206, an oral small molecule and allosteric Akt inhibitor, binds to the Akt protein through a site located in the pleckstrin-homology domain. The binding of MK-2206 induces a conformational change of Akt that prevents its localization to the plasma membrane, thus inhibiting its subsequent activation [1–5]. MK-2206 is a first-in-class highly selective inhibitor of all Akt isoforms, which is active in several human cancer models through a number of possible mechanisms, including the induction of autophagy and apoptosis in glioma cells [1–5]. As an anticancer agent, MK-2206 is being tested in adult tumors [6–12] and in a spectrum of pediatric tumors [13] both in vitro and in vivo. The effect of MK-2206 against glioma cells has been confirmed in vitro [14]. In addition, a recent clinical trial investigated the use of MK-2206 in patients with advanced solid tumors [15]. A more complete understanding of the mechanisms governing MK-2206 sensitization is required to facilitate its therapeutic use in patients with cancer. Identifying the mechanism(s) underlying cell sensitization to MK-2206 would be an important step in the development of new treatment methods for pharmacological cancer.

Salinomycin (Sal) was originally used to eliminate bacteria, fungi, and parasites [16, 17]. More recently, this drug has been exploited to inhibit the growth of tumor stem cells and chemoresistant cancer cells [18–20]. Sal also functions as an efflux pump p-glycoprotein (P-gp) inhibitor [21, 22] and is considered to be a potential anticancer drug for cancer chemoprevention. Sal, a polyether ionophore antibiotic isolated from* Streptomyces albus*, has been shown to kill cancer stem cells in different types of human cancers [23]. The ionophore involves various mechanisms, including inhibition of ABC transporters and oxidative phosphorylation [23]. In addition, Sal can overcome radiation resistance via inhibition of the Wnt/beta-catenin signaling pathway [23]. Sal can promote both cytoplasmic and mitochondrial potassium efflux and stimulate the differentiation of cancer stem cells [23]. Additionally, Sal sensitizes cancer cells to doxorubicin, etoposide, radiation, and antimitotic drugs [22, 24, 25]. Various Sal-sensitization mechanisms for cancer have also been investigated [26–28].

In the present study, we investigated whether cotreatment of Sal would sensitize cancer cells to MK-2206. We further analyzed whether the cotreatment influenced the activation status or levels of various signaling proteins of the PI3K/Akt/mTOR pathway.

## 2. Materials and Methods 

### 2.1. Reagents

Sal was purchased from Sigma-Aldrich (St. Louis, MO). MK-2206 was supplied by Selleckchem (Houston, TX). LY294002 was supplied by Calbiochem (Bellerica, MA).

### 2.2. Antibodies

Antibodies against Akt, phosphorylated Akt, PI3K, phosphorylated PDK1, phosphorylated TSC2, phosphorylated GSK3*β*, phosphorylated p70S6K, phosphorylated 4EBP1, mTOR, PTEN, FOXO1, PCNA, and cleaved poly ADP ribose polymerase (C-PARP) were from Cell Signaling Technology (Danvers, MA). Antibodies against glyceraldehyde 3-phosphate dehydrogenase (GAPDH), survivin, CDK4, and pRb were from Santa Cruz Biotechnology (Santa Cruz, CA). Antibodies against phosphorylated mTOR and phosphorylated PTEN were from Abcam (Cambridge, UK). Antibody against Cyclin D1 was from Biosource (Camarillo, CA).

### 2.3. Cell Culturing

Hs578T breast cancer cells were obtained from the Korean Cell Line Bank (Seoul, South Korea) and were previously used [22, 24–27, 29]. Human oral squamous carcinoma KB cell line was previously described [26, 30]. All cell lines were cultured in RPMI 1640 containing 10% fetal bovine serum, 100 U/mL penicillin, and 100 *μ*g/mL streptomycin (WelGENE, Daegu, South Korea).

### 2.4. Western Blot Analysis

Total cellular proteins were extracted using a previously described trichloroacetic acid (TCA) method [22, 24–27]. Briefly, cells grown in 60 mm dishes were washed three times with 5 mL PBS. Next, 500 *μ*L of 20% trichloroacetic acid (TCA) was added to each plate. The cells were then dislodged by scraping and were transferred to Eppendorf tubes. Proteins were pelleted by centrifugation for 5 min at 3000 rpm and resuspended in 1 M Tris-HCl (pH 8.0) buffer. The total protein concentrations were estimated. The proteins were resolved by sodium dodecyl sulfate-polyacrylamide gel electrophoresis (SDS-PAGE) and subjected to Western blot analysis as previously described [22, 24–27].

### 2.5. Fluorescence-Activated Cell Sorting (FACS) Analysis

FACS analysis was performed as previously described [22, 24–27]. Cells were grown in 60 mm dishes and treated with the indicated drugs for the prescribed times. The cells were then dislodged by trypsin and pelleted by centrifugation. The pelleted cells were washed thoroughly with PBS, suspended in 75% ethanol for at least 1 h at 4°C, washed again with PBS, and resuspended in a cold propidium iodide (PI) staining solution (100 *μ*g/mL RNase A and 50 *μ*g/mL PI in PBS) for 40 min at 37°C. The stained cells were analyzed for relative DNA content using a FACSCalibur flow cytometry system (BD Bioscience, Franklin Lakes, NJ). We performed more than two independent tests.

### 2.6. Hoechst Staining

The tests were used to identify nuclear disruption, an indicator of apoptosis. Briefly, cells in 6-well plates were treated with the indicated drugs and incubated for 24 h, 48 h, or 72 h at 37°C. Cells were then incubated with 9.4 *μ*M Hoechst 33258 (Sigma-Aldrich, St. Louis, MO) for 30 min in the dark at 37°C before image acquisition. The medium was removed, and the cells were washed twice with PBS. Stained cells were subsequently examined using an inverted fluorescence microscope. We performed more than two independent tests.

## 3. Results

### 3.1. Higher Concentration of Sal Reduced Both pAkt and Total Akt in MK-2206-Treated Cells

**Figure 1:**
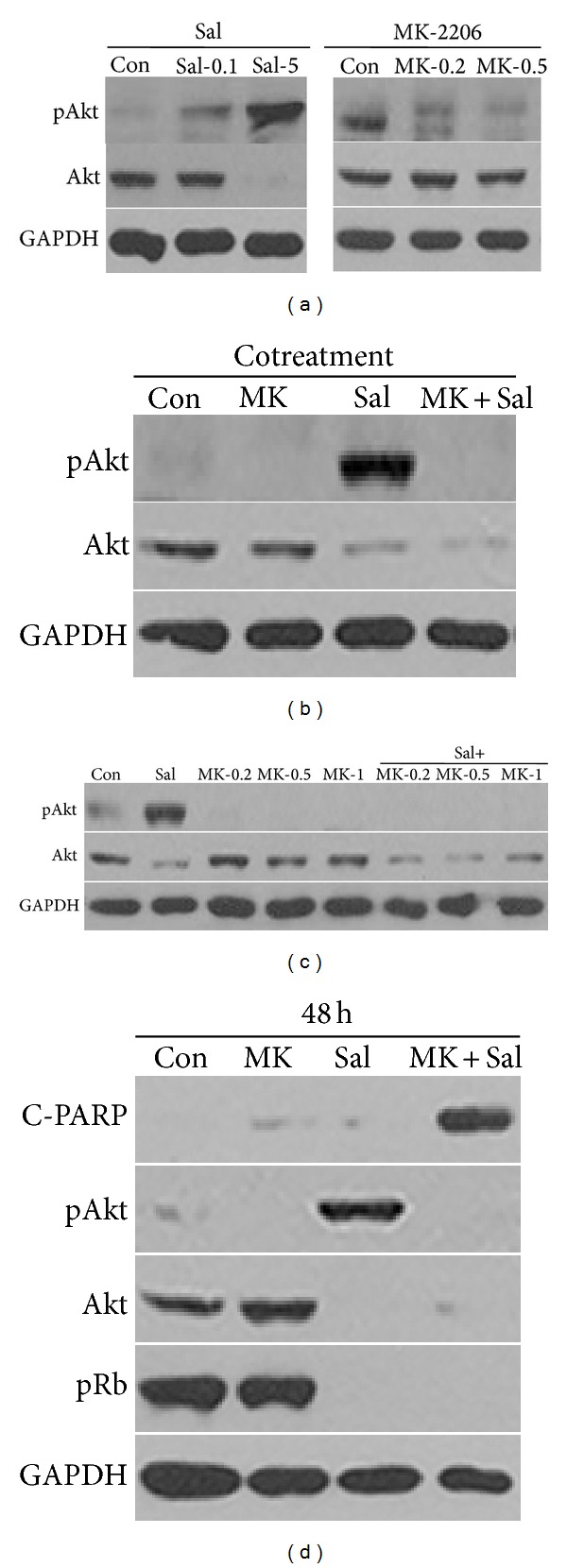
High concentration of Sal reduced pAkt and total Akt levels in MK-2206-treated cells. (a) Hs578T cell extracts were collected at 24 h after treatment with 0.1 *μ*M Sal (Sal-0.1), 5 *μ*M Sal (Sal-5), 0.2 *μ*M MK-2206 (MK-0.2), 0.5 *μ*M MK-2206 (MK-0.5), or DMSO (Con). (b) Hs578T cell extracts were collected at 24 h after treatment with 0.5 *μ*M MK-2206 (MK), 5 *μ*M Sal (Sal), 0.5 *μ*M MK-2206 with 5 *μ*M Sal (MK + Sal), or DMSO (Con). (c) Hs578T cell extracts were collected at 24 h after treatment with 5 *μ*M Sal (Sal), 0.2 *μ*M MK-2206 (MK-0.2), 0.5 *μ*M MK-2206 (MK-0.5), 1 *μ*M MK-2206 (MK-1), 5 *μ*M Sal with 0.2 *μ*M MK-2206 (Sal + MK-0.2), 5 *μ*M Sal with 0.5 *μ*M MK-2206 (Sal + MK-0.5), 5 *μ*M Sal with 1 *μ*M MK-2206 (Sal + MK-1), or DMSO (Con). (d) Hs578T cell extracts were collected at 48 h after treatment with 1 *μ*M MK-2206 (MK), 5 *μ*M Sal (Sal), 1 *μ*M MK-2206 with 5 *μ*M Sal (MK + Sal), or DMSO (Con). The cells were used for Western blot analyses using antibodies against pAkt, Akt, C-PARP, pRb, and GAPDH.

The potential for Sal to sensitize MK-2206-treated Hs578T breast cancer cells has been investigated. As shown in Figure 1(a), Akt activation was increased by Sal, while increasing concentrations of Sal induced a reduction in total Akt protein levels. In contrast, increasing concentrations of MK-2206 did not reduce total Akt protein levels, but it reduced pAkt levels (Figure 1(a)). The effect of MK-2206 and Sal cotreatment on pAkt and total Akt was then tested in Hs578T breast cancer cells. As shown in Figure 1(b), cotreatment with Sal and MK-2206 reduced both Sal-induced pAkt and total Akt protein levels, suggesting that combining MK-2206 and Sal treatments may reduce both pAkt and total Akt levels.

Dose and time dependence of the cotreatment effect on both pAkt and total Akt levels were further analyzed. As described in Figure 1(c), a low dose of MK-2206 can induce the reduction of both pAkt and total Akt levels in Sal-treated cells. Furthermore, the effect observed after 48 h of cotreatment was similar to the effect observed after 24 h of cotreatment (Figure 1(d)). C-PARP production was increased by MK-2206 and Sal cotreatment (Figure 1(d)), suggesting that the sensitization involved apoptosis. A reduction of pRb levels by the cotreatment was also observed, suggesting that the sensitization involved other cell cycle-related proteins. Collectively, our results indicated that Sal treatment can increase the sensitivity of cancer cells to MK-2206 by reducing total Akt protein levels.

### 3.2. Cotreatment with Sal and MK-2206 Increased Apoptosis

**Figure 2:**
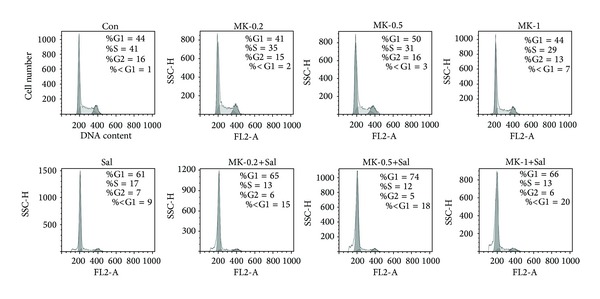
Cotreatment with MK-2206 and Sal increased pre-G1 regions in a dose-dependent manner. Hs578T cells were grown on 60 mm diameter dishes and treated with 0.2 *μ*M MK-2206 (MK-0.2), 0.5 *μ*M MK-2206 (MK-0.5), 1 *μ*M MK-2206 (MK-1), 5 *μ*M Sal (Sal), 0.2 *μ*M MK-2206 with 5 *μ*M Sal (MK-0.2 + Sal), 0.5 *μ*M MK-2206 with 5 *μ*M Sal (MK-0.5 +Sal), 1 *μ*M MK-2206 with 5 *μ*M Sal (MK-1 + Sal), or DMSO (Con). After 72 h, FACS analysis was performed as described in “Materials and Methods.”

**Figure 3:**
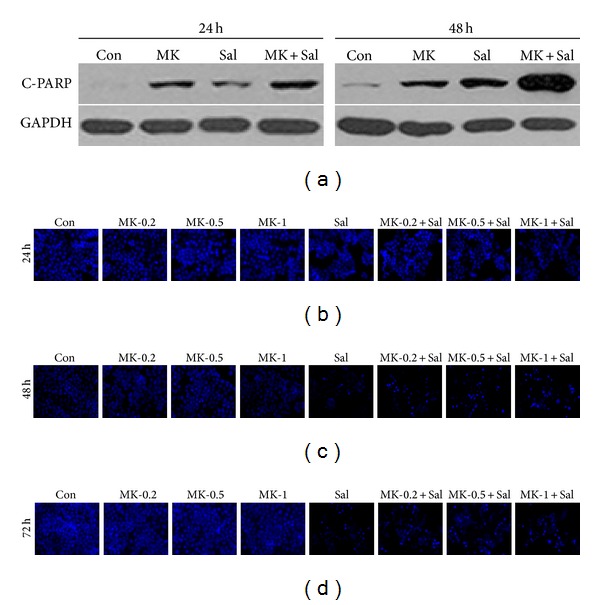
Cotreatment with MK-2206 and Sal increased apoptosis in a time-dependent manner. (a) Hs578T cell extracts were collected at 24 h or 48 h after treatment with 0.5 *μ*M MK-2206 (MK), 5 *μ*M Sal (Sal), 0.5 *μ*M MK-2206 with 5 *μ*M Sal (MK + Sal), or DMSO (Con). The cells were used for Western blot analyses using antibodies against C-PARP and GAPDH. (b–d) Hs578T cells were grown on 6-well plates and treated with 0.2 *μ*M MK-2206 (MK-0.2), 0.5 *μ*M MK-2206 (MK-0.5), 1 *μ*M MK-2206 (MK-1), 5 *μ*M Sal (Sal), 0.2 *μ*M MK-2206 with 5 *μ*M Sal (MK-0.2 + Sal), 0.5 *μ*M MK-2206 with 5 *μ*M Sal (MK-0.5 + Sal), 1 *μ*M MK-2206 with 5 *μ*M Sal (MK-1 + Sal), or DMSO (Con). After 24 h, 48 h, or 72 h, all cells were then stained with Hoechst as described in “Materials and Methods.” The stained cells were subsequently examined using an inverted fluorescence microscope with a 32x objective lens.

Cotreatment with Sal and MK-2206 increased pre-G1 regions in a dose-dependent manner (Figure 2), suggesting that the cotreatment with Sal led to an increase in the apoptosis of MK-2206-treated cells. In order to test whether the sensitization effect of the cotreatment was time dependent, we tested the time dependency of C-PARP production. As shown in Figure 3(a), when compared to the single treatments with MK-2206 or Sal, C-PARP production increased in a time-dependent manner when the cancer cells were cotreated with MK-2206 and Sal. To confirm these results, we performed Hoechst staining, which revealed marked morphological changes in cotreated cancer cells, consistent with apoptosis such as condensation of chromatin and nuclear fragmentation (Figures 3(b)–3(d)). Collectively, the data indicated that cotreatment with Sal increased the apoptosis of MK-2206-treated cancer cells in a dose- and time-dependent fashion.

### 3.3. Cotreatment with MK-2206 Reduced Sal-Activated GSk3*β*, TSC2, and 4EBP1

**Figure 4:**
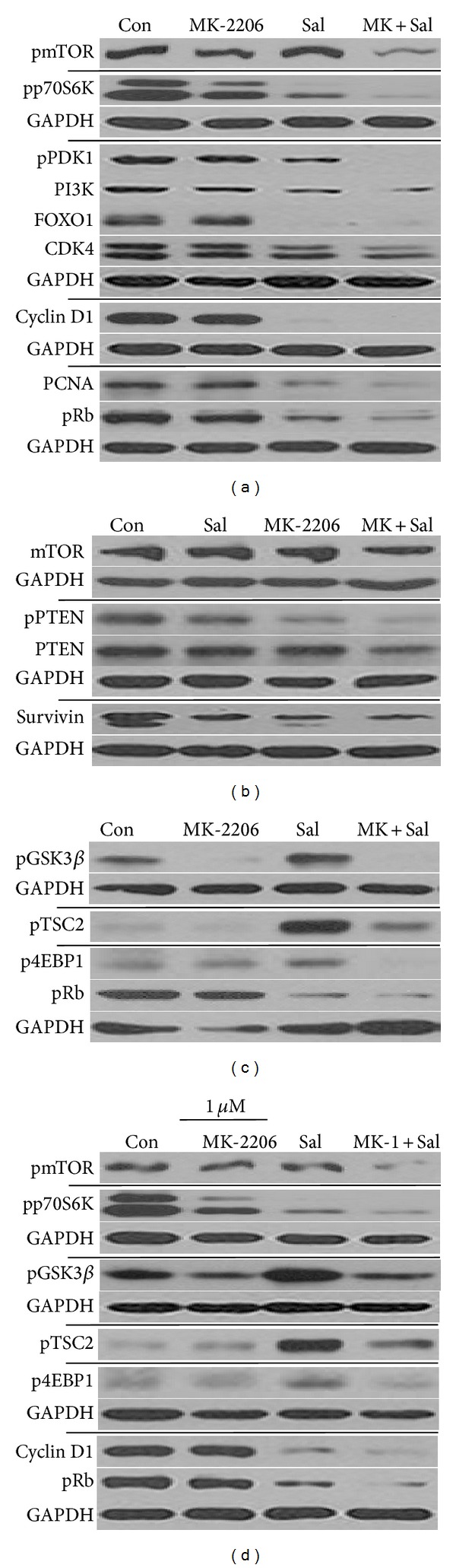
Cotreatment with MK-2206 reduced the levels of p70S6K, mTOR, and PDK1 activated forms and reduced Sal-activated GSk3*β*, TSC2, and 4EBP1. (a–c) Hs578T cell extracts were collected at 24 h after treatment with 0.5 *μ*M MK-2206, 5 *μ*M Sal (Sal), 0.5 *μ*M MK-2206 with 5 *μ*M Sal (MK + Sal), or DMSO (Con). The cells were used for Western blot analyses using antibodies against phosphorylated mTOR, mTOR, phosphorylated p70S6K, phosphorylated PDK1, phosphorylated GSK3*β*, phosphorylated TSC2, phosphorylated 4EBP1, phosphorylated PTEN, PTEN, PI3K, FOXO1, Survivin, Cyclin D1, CDK4, PCNA, pRb, and GAPDH. (d) Hs578T cell extracts were collected at 24 h after treatment with 1 *μ*M MK-2206, 5 *μ*M Sal (Sal), 1 *μ*M MK-2206 with 5 *μ*M Sal (MK-1 + Sal), or DMSO (Con). The cells were used for Western blot analyses using antibodies against phosphorylated mTOR, phosphorylated p70S6K, phosphorylated GSK3*β*, phosphorylated TSC2, phosphorylated 4EBP1, Cyclin D1, pRb, and GAPDH.

We further analyzed whether the cotreatment influenced the activation status or levels of the signaling proteins that function upstream and downstream of the Akt pathway. In this study, the major proteins of the PI3K/Akt/mTOR pathway, mTOR, p70S6K, PDK1, PI3K, GSK3*β*, TSC2, 4EBP1, and PTEN were tested [26, 31, 32]. MK-2206 single treatment did not affect pmTOR, pPDK1, PI3K, mTOR, and PTEN levels but reduced both pp70S6K and pPTEN (Figures 4(a) and 4(b)). Phospho-mTOR, pp70S6K, pPDK1, PI3K, pPTEN, and PTEN levels were further reduced in cancer cells cotreated with Sal and MK-2206, when compared to cancer cells treated with either MK-2206 or Sal alone (Figures 4(a) and 4(b)). Interestingly, as previously observed for pAkt (Figure 1(a)), the high concentration of Sal also increased pGSK3*β*, pTSC2, and p4EBP1 (Figure 4(c)). This effect of Sal was reduced or abolished by MK-2206 cotreatment (Figure 4(c)), suggesting that the activation of the PI3K/Akt/mTOR signaling pathway can be effectively reduced by MK-2206 cotreatment. Similar results were observed in the presence of high concentration of MK-2206 (Figure 4(d)). In conclusion, the cotreatment sensitization mechanism involved an effective reduction of various activated proteins belonging to the PI3K/Akt/mTOR pathway.

Since the PI3K/Akt/mTOR pathway is involved in proliferation and survival signals [26, 31, 32], we also tested whether the level of cell cycle- and proliferation-related proteins (FOXO1, CDK4, Cyclin D1, PCNA, and pRb) was reduced. MK-2206 single treatment did not affect these proteins (Figures 4(a) and 4(b)), whereas Sal treatment induced a reduction in most of them (Figures 4(a) and 4(b)). Cotreatment with Sal and MK-2206 had similar effects as Sal single treatment on cell cycle- and proliferation-related proteins (Figures 4(a) and 4(b)). It suggests that Sal cotreatment induced the reduction of cell cycle- and proliferation-related proteins in MK-2206-treated cells. In case of survivin, MK-2206 single treatment reduced protein levels in a manner similar to Sal single treatment (Figure 4(b)).

**Figure 5:**
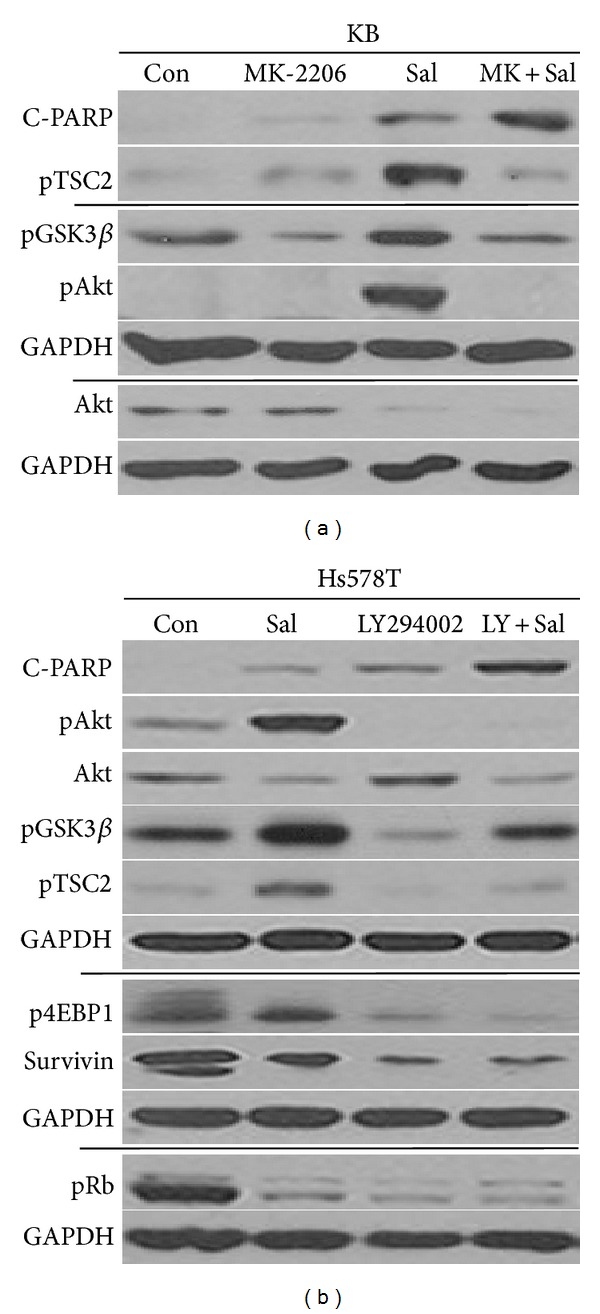
Sal and MK-2206 cotreatment sensitization mechanism is also conserved in KB cancer cell line. (a) KB cell extracts were collected at 24 h after treatment with 0.5 *μ*M MK-2206, 5 *μ*M Sal (Sal), 0.5 *μ*M MK-2206 with 5 *μ*M Sal (MK + Sal), or DMSO (Con). (b) Cotreatment with LY294002 reduced Sal-activated GSk3*β*, TSC2, and 4EBP1. Hs578T cell extracts were collected at 24 h after treatment with 5 *μ*M Sal (Sal), 20 *μ*M LY294002, 5 *μ*M Sal with 20 *μ*M LY294002 (Sal + LY), or DMSO (Con). The cells were used for Western blot analyses using antibodies against C-PARP, phosphorylated TSC2, phosphorylated GSK3*β*, phosphorylated Akt, Akt, phosphorylated 4EBP1, Survivin, pRb, and GAPDH.

Other cell lines were tested to assess whether a similar sensitization mechanism could be observed. KB cell line presented an increase in C-PARP production when cotreated with MK-2206 and Sal (Figure 5(a)). As observed in Hs578T cells, pTSC2, pGSK3*β*, pAkt, and total Akt proteins were also reduced in KB cells cotreated with Sal and MK-2206 (Figure 5(a)), suggesting that Sal and MK-2206 cotreatment sensitization mechanism is also conserved in KB cancer cell line. However, future studies are warranted to determine whether this sensitization effect is observed in other cancer cell types.

### 3.4. Cotreatment with LY294002 Reduced Sal-Activated GSk3*β*, TSC2, and 4EBP1

Since MK-2206 is known to be an Akt inhibitor [1–5], we tested whether another Akt inhibitor LY294002 [26, 31, 32] also had similar effects. As shown in Figure 5(b), LY294002 and Sal cotreatment increased C-PARP production, suggesting that the cotreatment with LY294002 sensitized Sal-treated cells. In addition, as with MK-2206 cotreatment, the sensitization mechanism induced by LY294002 cotreatment involved the reduction of pAkt, total Akt, pGSK3*β*, pTSC2, and p4EBP1 (Figures 4(a)–4(c) versus Figure 5(b)). LY294002 single treatment reduced pRb levels (Figure 5(b)), whereas MK-2206 single treatment did not (Figures 4(a) and 4(c)), suggesting that MK-2206 is a more specific Akt inhibitor than LY294002. Collectively, our results indicated that the sensitization mechanism observed when Sal is combined with MK-2206 can be observed when combined with other Akt inhibitors.

## 4. Discussion

MK-2206 is a recently developed drug that targets Akt activation [1–5]. In this study, we attempted to identify ways to sensitize MK-2206-treated cells or ways to overcome MK-2206-resistance in cancer cells. We hypothesized that MK-2206-treated cancer cells can be sensitized if total Akt protein levels are reduced. We determined that both total Akt and pAkt levels could be reduced by Sal and MK-2206 cotreatment. We also demonstrated that a relatively low dose of MK-2206 is enough to reduce both pAkt and total Akt levels in Sal and MK-2206 cotreated cancer cells. This finding suggests that MK-2206 toxicity can be reduced by combining the treatment with Sal in future clinical trials. Sensitization of cancer cells to various anticancer drugs and radiation by cotreatments with Sal has been demonstrated [22, 24, 25]. However, the sensitization of cancer cells to a specific molecular-targeting drug using Sal has yet to be determined. To the best of our knowledge, our work is the first report of the sensitization of MK-2206-treated cells by Sal cotreatment. This suggests that Sal may also be useful in combination with various specific molecule-targeting drugs. Furthermore, Sal was shown to reduce total Akt protein levels. In conclusion, high concentrations of Sal can be used to reduce total Akt levels or prevent the generation of its activated form, pAkt. Further studies are warranted to understand the mechanism(s) by which Sal reduces total Akt protein levels. These future studies include measuring Akt mRNA levels, Akt protein stability, and Akt protein translation. The signaling pathways involved in the reduction of total Akt levels by Sal should also be investigated. Since Sal has been shown to sensitize resistant cancer cells or cancer stem cells, cotreatments using MK-2206 and Sal could also be applied to these cell types.

We further analyzed the activation status or levels of the PI3K/mTOR/Akt pathway signaling proteins. Sal reduced pmTOR, pp70S6K, pPDK1, PI3K, pPTEN, and PTEN protein levels in MK-2206-treated cells. Interestingly, we found that high concentration of Sal increased pAkt, pGSK3*β*, pTSC2, and p4EBP1. The increased activation of these proteins by Sal was reduced by MK-2206 cotreatment, suggesting that various Sal-activated PI3K/Akt/mTOR pathway proteins are effectively reduced by MK-2206 cotreatment. In addition, Sal treatment reduced levels of proliferation- and survival-related proteins such as FOXO1, CDK4, Cyclin D1, PCNA, and pRb in MK-2206-treated cells. Therefore, cotreatment with Sal and MK-2206 allows the reduction of two types of protein families: reduction of Sal-activated proteins by MK-2206 and reduction of cell cycle- and proliferation-related proteins by Sal.

Our data indicated that cotreatment-induced sensitization mechanism involves apoptosis, since an increase in C-PARP production and pre-G1 region was detected and confirmed by Hoechst staining. The apoptotic effect of the cotreatment was observed for a long period of time after cotreatment, suggesting that the initial treatment could be effective for long periods of time. It also suggests that the number of cotreatments could be reduced when used in clinical trials. The cotreatment with MK-2206 and Sal sensitized two different cancer cell lines, Hs578T breast cancer and KB oral squamous cancer cell lines, suggesting that the sensitization mechanism of the cotreatment is generally conserved in different cancer cell lines. However, future studies are warranted to determine whether this sensitization effect is observed in other cancer cell types. LY294002, another Akt inhibitor [26, 31, 32], was also found to reduce total Akt protein as well as activated Akt, pGSK3*β*, pTSC2, and p4EBP1 in Sal-treated cells. The fact that the sensitization effect is observed when combining Sal with either LY294002 or MK-2206 suggests that this effect may be conserved among Akt inhibitors. These results suggest that various Akt inhibitors could be combined with Sal for sensitization.

In summary, our results could help determine the potential clinical use of Sal for MK-2206-treated cancer patients. The present study also enhances our understanding of Sal-sensitization mechanisms. Our findings may contribute to the development of MK-2206-based therapies for patients.

## Abbreviations

Sal: Salinomycin
MK: MK-2206
LY: LY294002
PI3K: Phosphatidylinositol 3-kinase
C-PARP: Cleaved poly ADP ribose polymerase
DMSO: Dimethylsulfoxide
FACS: Fluorescence-activated cell sorting
FBS: Fetal bovine serum
TCA: Trichloroacetic acid
PBS: Phosphate-buffered saline
SDS-PAGE: Sodium dodecyl sulfate-polyacrylamide gel electrophoresis
RT: Room temperature
mTOR: Mammalian target of rapamycin.
